# COVID-19-Related Mortality amongst Physicians in Italy: Trend Pre- and Post-SARS-CoV-2 Vaccination Campaign

**DOI:** 10.3390/healthcare10071187

**Published:** 2022-06-24

**Authors:** Alberto Modenese, Tom Loney, Fabriziomaria Gobba

**Affiliations:** 1 Department of Biomedical, Metabolic and Neural Sciences, University of Modena and Reggio Emilia, 41125 Modena, Italy; f.gobba@unimore.it; 2 College of Medicine, Mohammed Bin Rashid University of Medicine and Health Sciences, Dubai P.O. Box 505055, United Arab Emirates; tom.loney@mbru.ac.ae

**Keywords:** SARS-CoV-2, vaccination campaign, COVID-19, healthcare workers, occupational risk

## Abstract

Globally, there has been a high burden of COVID-19-related mortality amongst physicians and other healthcare workers during the ongoing SARS-CoV-2 pandemic. Fortunately, anti-COVID-19 vaccination campaigns have helped to protect frontline workers and reduce COVID-19-related mortality amongst this occupational group. We analyzed COVID-19-related mortality data for doctors in Italy and compared the crude mortality rate between March–May 2020 (i.e., the beginning of the pandemic in Italy, with the highest rates of COVID-19-related deaths) and the same time period in March–May 2021 (high vaccination coverage amongst Italian physicians). The mortality rate was 12 times higher in March–May 2020 compared to the same time period after the start of the Italian vaccination campaign. Moreover, there was a strong inverse correlation between the number of deaths and the cumulative number of vaccine doses administered in the Italian population. Although non-pharmaceutical interventions, virus evolution and environmental factors probably had an effect, our analysis clearly supports the hypothesis that the vaccination campaign helped to protect Italian physicians and reduce COVID-19-related mortality. The latest available death trends from September to October 2021 for both physicians and the general population are also in favor of the need for the third vaccine dose, currently underway for the majority of the population at risk.

## 1. Introduction

The impact of the SARS-CoV-2 pandemic on the lives of healthcare workers (HCW) is well known, as they have been in the frontline to assist COVID-19 patients. Specifically, HCWs have been exposed to a high degree of job stress, resulting in a high prevalence of burn-out related to working conditions, including very long working hours [1,2,3]. Due to the nature of their job, HCWs have a higher risk of acquiring the infection compared to the general public, and this has resulted in a considerable number of health personnel experiencing severe consequences after COVID-19. These include medical complications and long-term hospitalizations, problems after the resolution of the acute infection (i.e., long COVID) with issues in returning to work and, unfortunately, an extremely relevant number of work-related deaths [4,5,6]. Reports on COVID-19-related deaths among HCW at the beginning of the pandemic indicated that Italy was the country with the highest number of reported deaths among HCW. At the beginning of April 2020, SARS-CoV-2 was reported as the cause of death for over 100 HCW, nearly half of whom were in Italy, while at the end of April, the number had nearly doubled, with 151 doctors and more than 40 nurses dying from COVID-19-related illness in Italy [5,7]. It should be noted that elderly retired HCW and voluntary HCW in Italy were recruited to increase the healthcare workforce capacity, often with a relevant shortage of PPE [5,7,8]. Ing et al., comparing data updated to the middle of April 2020 from various countries, showed that Italy was ranked first for the number of deaths among physicians, with 121 cases, followed by Iran with 43 deaths, the Philippines with 21, Indonesia with 17, China with 16, Spain with 12, the USA with 12, the UK with 11, France with 7 and other countries with less than three reported deaths [9]. More recent reports indicated increased deaths with the protracting of the pandemic: the number of deaths rose up to 108 in October 2020 [10] and 132 in November in the United States [11], and to 44 in the United Kingdom in February 2021 [12]. Recent available reports show even higher numbers, with 3607 HCW, 17% of which were physicians, who died in the US after one year of the pandemic in April 2021 [13], and with Asian data showing that a total of 545 doctors died in Indonesia, of which 117 occurred in one month only, i.e., in July 2021 [14]. Moreover, in the same month, the Indian Medical Association reported 748 COVID-19-related deaths among doctors during the first wave of the pandemic and 798 during the second wave [15].

The general aim of the present work was to update the reporting on COVID-19-related mortality amongst Italian physicians during nearly twenty months of the pandemic. In addition, we evaluated the crude mortality rate according to sex, age, and different medical specialties, as well as the changes in death trends before and after the start of the vaccination campaign in Italy on the 27 December 2020 [16]. The first vaccine approved in Italy was the Comirnaty (Pfizer-BioNtech), immediately followed by another mRNA vaccine, i.e., the COVID-19 Moderna Vaccine (Moderna) and then by the Vaxzevria (AstraZeneca) vaccine at the end of January 2021 and by the Janssen (Johnson & Johnson) vaccine in March 2021. Currently, the Nuvaxovid (Novavax) vaccine is also approved, but it was not at the time of the present study [16,17]. The vaccination campaign in Italy firstly involved all the healthcare workers and the most vulnerable groups of the population (i.e., older subjects hospitalized in nursing homes and subjects with pathologic conditions determining a particular susceptibility to severe COVID-19-related health consequences). These subjects had been vaccinated mainly in the period January–March 2021, with two doses of mRNA vaccine [16,17]. On the other hand, the majority of the general population had been vaccinated after the beginning of March 2021, with priority based on age (i.e., the older groups first, then the younger) [17]. Based on the data from the Italian National Institute of Health, at the beginning of May 2021, 61% of the population had received at least one dose of the Comirnaty vaccine, 31% at least one dose of the Vaxzevria vaccine, 8% at least one dose of the Moderna vaccine and <1% of the Janssen vaccine (which required a single dose) [18]. Considering the third vaccine dose in Italy, the administration of the booster started at the end of September 2021 [17], and therefore it was not considered in the present study.

According to these premises, the specific objectives of our work were to:

*i*. identify whether there were differences in the mortality rates of Italian physicians compared to those of the general public during the same time periods, indicating possible changes in the occupational risk with the evolution of the pandemic;

*ii*. evaluate additional work-related factors indicating an occupational risk for COVID-19-related deaths, e.g., according to the specific jobs performed by the physicians based on their medical specialty;

*iii*. appreciate the protective effect of anti-SARS-CoV-2 vaccinations on mortality rates, as the campaign started earlier for physicians compared to the general population; moreover, there are differences in the vaccines applied in the two populations: physicians were more likely to be vaccinated with mRNA vaccines only, while the general population was more likely to be vaccinated with only one dose and to be unvaccinated compared to physicians;

*iv*. confirm other factors, in addition to vaccination, possibly related to the trends in COVID-19-related mortality rates in Italy, as, e.g., season of the year and improvements in the prevention and control strategies, based on the long observation period of the study (i.e., twenty months).

## 2. Materials and Methods

We retrieved the date of death and the medical specialty from the publicly available data on COVID-19-related deaths amongst Italian physicians during the period from 11 March 2020 (first date of reported death) up to the 7 October 2021, published on the official webpage of the Italian Federation of the Colleges of Physicians and Dentists (FNOMCEO) [19]. The FNOMCEO website has constantly updated mortality statistics amongst Italian physicians, including dentists, since the beginning of the pandemic up to the present [19]. Age and sex, not included in the FNOMCEO webpage, were obtained from the registry office of the Italian physicians [20]. Retired physicians are included in the FNOMCEO database and it is known that many of the formerly retired physicians returned to clinical work to serve the community during the COVID-19 pandemic. When this information was not specifically available on the website, we established an arbitrary cut-off age of 75 years (seven years after the official current retirement age of 68 years) to consider a physician as most likely not yet directly involved in health assistance activities. When no information on retirement or age was available, we kept the data of the physicians’ deaths in our dataset.

In order to compare the study population with the overall population of Italian physicians, we considered a specific FNOMCEO report, with data on age and gender distributions of all the Italian physicians, lastly updated on 2 March 2021 [21]. For the total number of general practitioners operating in Italy, we consulted data elaborated from the “Health Atlas” (i.e., “Atlante Sanità”), which is one of the main Italian healthcare databases [22]. We then analyzed possible statistically significant differences in the distribution by age group (<50 vs. ≥50 years old), sex and medical specialty (general practitioners vs. other specialties), comparing deceased physicians with the whole doctors’ population, performing Pearson’s chi-squared tests. Differences in mean age of the physicians who died according to their sex, medical specialty and month of death have been evaluated by application of Student’s *t*-test for independent samples and ANOVAs.

We then reconstructed the cumulative frequency of COVID-19-related deaths among physicians in the period 11 March 2020–7 October 2021 and we compared these data with the number of deaths that occurred in the general Italian population during the same period, available from the Italian National Institute of Health [23]. For a better comparison of the numbers, we reconstructed the mortality rates based on the above-mentioned latest available FNOMCEO report on the total numbers of physicians in Italy [21] and on the latest census of the Italian population available through the National Institute of Statistics (ISTAT) [24]. We also compared the main demographic characteristics of the death cases among physicians and the general public, considering their mean age at the time of death and the sex distribution, evaluating possible statistically significant differences by application, respectively, of Student’s t-test for independent samples and Pearson’s chi-squared test. Moreover, we evaluated by chi-square analysis the difference in the percentage distributions of death cases between physicians and the general public considering the total number of deaths that occurred before and after the start of the anti-SARS-CoV-2 vaccination campaign, setting as the starting month for the physicians January 2021, while for the general public, it was March 2021. Furthermore, in order to appreciate differences according to the season of the year, we established as “warm” months all the late spring, summer and early autumn months of both years 2020 and 2021 included in the analysis (i.e., May–October 2020 and 2021), while, as “cold” months, we considered late autumn, early spring and winter months (i.e., March–April 2020 and 2021, November–December 2020 and January–February 2021), and we evaluated the proportional death rates among the two groups of the population by Pearson’s chi-square test.

Finally, as almost all the physicians were vaccinated during the first few months of the campaign (i.e., January–February 2021), we compared the differences in the number of deaths during the same days in March–May 2020 (start of epidemic in Italy; no vaccinations) and March–May 2021, and with the cumulative numbers of vaccine doses administered in the Italian population [25]. Two doses of anti-SARS-CoV-2 vaccine were required in Italy, and HCW were a high-exposure priority group to receive vaccines. We compared the daily difference in cumulative deaths with the total number of vaccine doses administered 30 days before, in order to have an estimation of the possible protective effect of a full vaccination cycle, considering that the majority of the physicians were vaccinated with mRNA vaccines, requiring, at that time in Italy, 21 and 28 days between the first and the second dose, respectively, for the Comirnaty and the Moderna vaccines [16]. Pearson’s *r* coefficient was calculated to evaluate the bivariate linear correlation between the two continuous variables. Data cleaning and statistical analysis were conducted in Microsoft Office Excel 2016.

## 3. Results

### 3.1. Physicians’ Deaths in Italy from March 2020 to October 2021 by Sex, Age, and Medical Specialty

The first report of a death related to SARS-CoV-2 infection in an Italian physician was on the 11 March 2020. According to the official data of the FNOMCEO, there were 293 COVID-19-related deaths amongst Italian physicians between 11 March 2020 and 7 October 2021. Their mean age was 65.9 years old, and 94.9% of them were males. We observed no statistical differences between sexes considering the mean age at the time of death (Table 1). Comparing the study population with all Italian physicians, who totaled 459,884, with 57% males and 43% females, there was a highly significant difference in the sex distribution of the two groups in the chi-square test (χ^2^ = 171.65; *p* < 0.0001). Considering the age, 98.2% of the physicians who died were ≥50 years old, vs. 58.9% of all Italian doctors, also in this case with a highly significant difference (χ^2^ = 176.07; *p* < 0.0001).

The crude mortality rate for the whole physician population was 0.6‰. Considering the medical specialties involved, the majority of deaths occurred among general practitioners (GP), representing 41% of the total deaths, followed by dentists (7.5%), anesthetists (5.5%) and public health doctors and gynecologists (both with 3.1%). No statistical differences were observed in the mean age of the physicians grouped according to their medical specialty (Table 1). Comparing the proportion of general practitioners within the group of deceased physicians (i.e., 41.3%) with that registered within the whole population of Italian physicians, which was 10.9%, we observed a highly significant statistical difference in the chi-square test (χ^2^ = 278.56; *p* < 0.0001).

### 3.2. Time Trend of Physicians’ Deaths Compared to the General Population and Considering the Vaccination Campaign

Compared to the general population, as expected, the mean age of the deceased physicians was significantly lower (65.9 vs. 80.0 years, *p* < 0.0001). Moreover, the sex distribution of the death cases was significantly different between the two populations, with males representing 94.1% among physicians and 56.5% in the general public (χ^2^ = 175.29; *p* < 0.0001) (Table 2).

Nearly half of the physicians’ deaths (41.3%) were observed at the beginning of the SARS-CoV-2 epidemic in Italy, in the period between March and April 2020, and a third (31.7%) in the period November–December 2020. Lower numbers of deaths were reported after the beginning of the vaccination campaign (i.e., 27 December 2020) in the period January–February 2021. Specifically, there was a 37% difference in the number of deaths compared to the two months before the vaccination campaign. During March–April 2021, only 14 deaths were reported, compared to the 121 registered in the same period of the previous year. From May to October 2021, only four deaths were reported. Considering the start of the vaccination campaign in January for physicians and in March for the general population, it could be observed that 81.6% of the total deaths were reported before the vaccination campaign for physicians vs. 76.5% for the general public, and the difference was significant in the chi-square test (χ^2^ = 4.10; *p* = 0.04) (Table 2).

For both physicians and the general public, a low percentage of deaths, 9.9% and 14.3%, respectively, was reported during the Italian warm season compared to the colder months, both in 2020 and in 2021. This proportion of death cases during the warmer months was slightly lower for physicians compared to the general public, with a statistically significant difference (χ^2^ = 4.65; *p* = 0.03) (Table 2).

Observing the mortality data for the general population, it can be seen that during 2021, the bimonthly percentages for the total number of deaths remained quite high, compared to the percentages reported in the same period for physicians, until September–October 2021, when the two percentages were almost comparable (Table 2). In fact, in January–February 2021, 17.2% of the total COVID-19-related deaths were reported for the general public vs. 12.3% in physicians. Similarly, in March–April 2021, the crude mortality rate for the general population was 17.0% vs. only 4.8% in physicians. Lower but still significant percentages for the general population given the absolute size of the population were reported during May–June (4.1%) and July–August 2021 (1.1%), compared to 0% and 0.3% among physicians during the same time period (Table 2).

**Figure 1:**
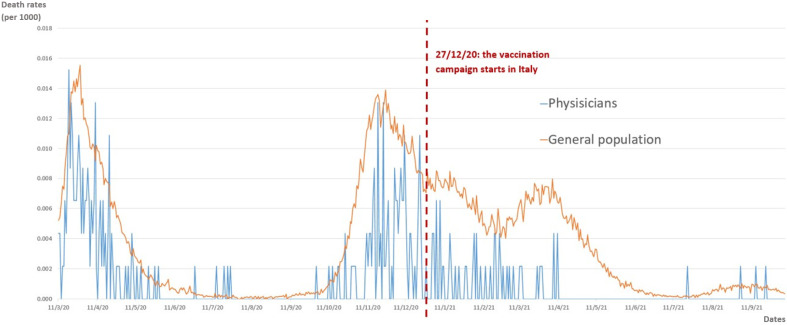
Time trend of COVID-19-related death rates per 1000 subjects among Italian physicians and the general public during the period March 2020–October 2021.

Figure 1 illustrates the time trend of the COVID-19-related death rates among Italian physicians and the whole population during the twenty months of the pandemic.

The peak of daily deaths was first reached among physicians on the 19th of March 2020, with seven doctors who died on this day, while, for the general population, the peak was reached after nine days, with 927 daily deaths. The curve of the daily deaths among physicians resembles the curve of the whole Italian population, approximately until the first ten days of January 2021, and then a relevant decrease in the doctors’ mortality rate was observed, with a significant deviation from the curve of the general public’s death trend up to the end of the summer 2021 (Figure 1).

**Figure 2:**
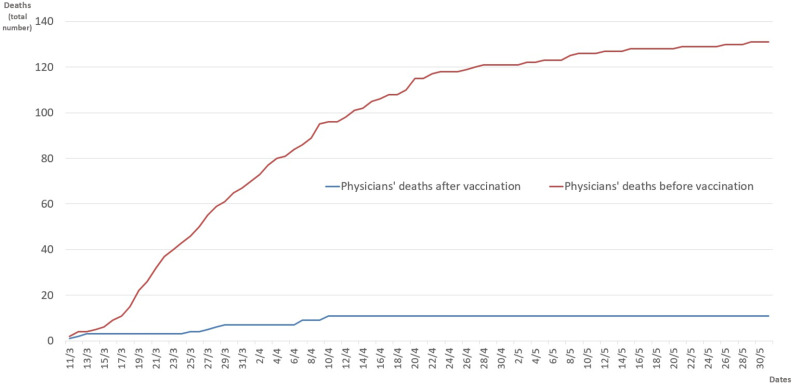
Comparison of the total number of COVID-19-related physicians’ deaths observed during the same period in March–May 2020 vs. 2021, i.e., before and after the start of the vaccination campaign in Italy.

In Figure 2, we compare the cumulative number of deaths that occurred during the same period, between the 11th of March and the 31st of May in the years 2020 and 2021, i.e., before and after the beginning of the vaccination program. In 2021, the curve of the total number of physicians’ deaths was almost flat from the beginning of April, with only a slight increase during March. On the contrary, the cumulative number of physicians’ deaths rapidly increased from March to the end of May 2020, with the total number of deaths becoming five times higher than the total deaths observed in 2021 on the 18 March, and reaching a number 10 times higher than the following year on the 21 March, maintaining this difference up to the end of May 2020 (Figure 2).

**Figure 3:**
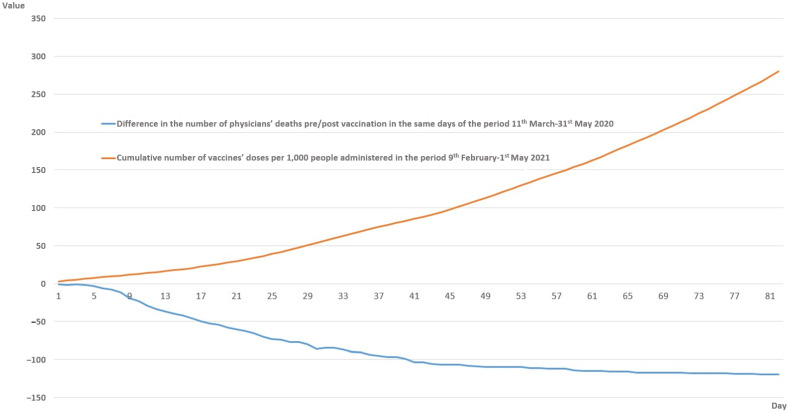
Difference in the cumulative number of COVID-19-related deaths amongst physicians between the same period in 2020 vs. 2021, from day 1 to day 82 (i.e., 11 March–31 May), and the number of vaccine doses administered in Italy in the previous 30 days of 2021 (i.e., 9 February–1 May 2021).

Finally, in Figure 3, an inverse correlation (r = −0.83) between the increasing difference in the cumulative number of physicians’ deaths between the same days in 2020 vs. 2021, from the 11 March to the 31 May, and the increasing number of vaccine doses administered in Italy can be observed. However, it should be noted that, as explained in the Section 2, the cumulative number of vaccine doses refers to 30 days before. The difference in the number of deaths rapidly increased up to 100 cases at the end of April, and then increased slowly up to 120 in May. On the other hand, the cumulative number of vaccine doses in Italy constantly increased during the observation period, with an upwards trend. During the first day (i.e., the 9 February 2021, which is the date that we compared with the deaths registered on the 11 March), we collected data of a total of 2.75 doses administered per 1000 people, reaching a value on the 1 May of 280.31 doses per 1000 inhabitants (Figure 3).

## 4. Discussion

The data reported here on the trend of COVID-19-related deaths amongst Italian physicians after the beginning of the anti-SARS-CoV-2 vaccination campaign confirm the relevant tribute paid in lives lost by the doctors [7,8,9,10,11,12,13,14,15], but also clearly provides further support to the important protective effect of the vaccines against the risk of death [26].

Considering the different medical specialties, the present article provides supporting evidence for our previous report, showing an increased risk in particular for general practitioners [8]. Moreover, other medical activities have been reported again with a higher number of deaths compared to others—in particular, among dentists, anesthetists, and cardiologists. The only difference with the previous report seems related to surgeons, for whom the percentage of deaths was not as high as in the previous investigation, which was limited to the end of April 2020 [8]: this may reflect an improvement in the SARS-CoV-2 risk prevention during surgical procedures, as well as the large reduction in the number of surgical interventions, particularly non-urgent ones, during the most severe period of the pandemic.

Regarding the age and sex characteristics of the physicians, the data indicated a mean age at the time of death of 66 years, with no significant differences between men and women, and no differences among the various medical specialties. The Italian National Institute of Health indicated that the mean age of the subjects dying from SARS-CoV-2 infection in Italy was 80 years, and the sex distribution of the death cases was 56.5% male and 43.5% female [27]. The lower mean age of the physicians might possibly be explained by the fact that we limited our analysis only to the working population, considering as an upper limit for the inclusion in the study an age of ≤75 years. The difference in the male/female ratio in our analysis might be partially explained by the observation that 95% of the deaths involved males. Firstly, this percentage should not be compared with the overall ratio reported in the Italian official data for the general population, but more appropriately with the ratios of the deaths that occurred in males vs. females in younger age groups, excluding the elderly. In this case, the ratio is approximately 70% males vs. 30% females in the age groups up to 79 years old, after which it becomes more homogeneous [27]. Another aspect that should be considered in interpreting our data is that the overall proportion of male vs. female physicians in Italy is 60% vs. 40%, but this ratio changes to 70% vs. 30% when considering only physicians ≥60 years old, while, in the Italian general population, women represent 51% of the population, but in the older groups, females reach percentages up to 64% above 85 years and to 80% above 95 years [28]. After these considerations, the data reported are in line with the higher risk of severe COVID-19 consequences known for elderly males [1,2,3], and it is particularly high, possibly because the majority of the older physicians who returned to work to help during the pandemic after retirement were male.

Analyzing the trend of the physicians’ deaths and the deaths that occurred in the general public, it can be seen that the mortality rates followed the same curve until the beginning of the anti-SARS-CoV-2 vaccination campaign in Italy, at the very end of 2020 (Figure 1). The highest mortality rates per 1000 subjects involved both physicians and the general public at the beginning of the pandemic, and are almost comparable, with a possible exception when observing the death rates during the first two months of the Italian epidemic, i.e., March–April 2020. During this period, 41% of the total deaths observed among physicians were reported, vs. 22% of the cumulative deaths in the general population. These data may reflect the high occupational risk of the healthcare personnel compared to the general public, particularly clear at the beginning of the pandemic, when insufficient information on the disease and on the most adequate protective measures was available and there was a scarcity of protection, considering the exponential spreading of the contagion. On the other hand, the data can also be related to the insufficient availability of diagnostic tests at the beginning of the pandemic, together with the extremely high impact of the first COVID-19 wave in Italy during a short period of time, overloading and saturating the healthcare system. According to this, it is unfortunately possible that not all the people who died with a suspected COVID-19 diagnosis had actually been tested for SARS-CoV-2 infection in March–April 2020, and this is more likely for the general population. It has been reported that, at the beginning of the pandemic in Italy, many subjects may have died at home, without hospitalization and confirmation of the diagnosis.

Another relevant indication of the high occupational risk of death due to COVID-19 for physicians is related to the fact that, while for the general population all the age classes are included in the analysis, for physicians we set an upper limit of 75 years old, but the death rates of the two groups are almost comparable until the start of the vaccination campaign. This observation suggests an increased risk for doctors, as younger adults are usually more protected against the severe consequences of COVID-19 compared to older ones, so that the similar mortality rates may lead to a higher incidence of SARS-CoV-2 infections among physicians [1,2,3,4,5,6]. Almost immediately after the start of the campaign, from the beginning of January, the curve of the death rate among physicians started to deviate from the curve of the general population, indicating an important protective effect after the first vaccine dose (Figure 1). The crude mortality rate for the doctors remained stable at approximately 20–30% of the rate observed for the general population during the period of November–December 2020 up until the beginning of April. During these months, the curve for the general public displayed a slight decrease during January and February, probably related to both the effects of a strict lockdown together with the first effects of the vaccinations administered not only to HCW but also to clinically vulnerable and elderly adults; it then started to increase in March and April. This may be explained by the interruption of the strictest lockdown measures, as well as insufficient availability of vaccine doses during the first few months of the campaign in Italy for the general public. Even if the effectiveness of the vaccination campaign is clear for both the groups, we observed a significant difference in the death rates of physicians vs. the general public after the start of vaccinations, with the campaign appearing slightly more effective in reducing death rates among the physicians. This might be related to the differences in the vaccination campaign between the two groups, but also to an improvement in infection control practices and personal protective measures among the well-prepared and trained doctors after one year of the pandemic. From mid-April up to October 2021, there were only four deaths among physicians, and also the curve for the general public started to decrease to a lower level, close to zero during the months of July and August 2021, when the proportion of fully vaccinated adults in Italy exceeded 70% of the total population [29]. In addition, increased time spent outdoors during the summer, as observed also in 2020, might have contributed to lowering the crude rate of COVID-19-related mortality [30,31]. These suggestion is supported by our analysis observing a relevant decrease in the death cases for both physicians and the general public in the warm vs. the cold Italian season. Nevertheless, also in this case, a slightly significant difference can be appreciated when comparing the two groups, confirming a stronger effect in the doctors’ group, therefore suggesting the role of additional prevention and control measures. Firstly, the vaccination campaign started earlier and was more homogeneous for physicians. The four death cases observed among physicians during August and September, as well as the slight increase in death rates for the general public after the second half of August, provide support for the role of seasonality, slowly moving from warmer to colder months, but also highlight the need for the booster vaccine dose, with its administration beginning in Italy at the end of September 2021 and currently ongoing for the majority of the population at risk.

In order to further explore the effect of the vaccination campaign on the COVID-19-related mortality rates amongst physicians, we first compared the total number of deaths between the same days during the period March–May in 2020 vs. 2021 (Figure 2) and then we correlated the difference in the absolute number of deaths with the total number of vaccine doses administered 30 days before in 2021 (Figure 3). It can be observed that during these three months, the total number of physicians who died in 2020 compared to 2021 was around twelve times higher, and that there was a strong inverse correlation between the cumulative number of vaccine doses administered and the increasing difference in the total number of deaths (Figure 3). As the dates are the same in 2020 compared to 2021, the role of possible environmental factors involved in reducing the SARS-CoV-2 infection incidence can be supposed to be almost null [30,31]. Moreover, in Italy, during these months in both 2020 and 2021, very strict lockdown measures were taken [31]. Accordingly, the difference in death rates might be explained by the effect of the vaccination campaign, together with better knowledge of the risk of infection, greater awareness of SARS-CoV-2 preventive measures after one year of the pandemic and changes in the virulence of the predominant viral variant circulating in the Italian population over time. Nonetheless, the protective effect of the vaccines on the risk of COVID-19-related death for doctors is not only related to the receipt of the vaccination, but also to a more general effect related to the increased proportion of the vaccinated population, reducing the transmission of SARS-CoV-2 from person to person.

Our study has several limitations; firstly, it is an ecological study not able to evaluate any causal relationship according to the statistical methods applied, but only observing the trends of a phenomenon, suggesting possible associations and correlations. Secondly, assumptions have been made, as described in detail in the Section 2, both related to the study of the physicians’ deaths as well as to the collection of the vaccine data. For the former, the main assumption was related to the cut-off age (≤75 years) that we established for inclusion in the study. We arbitrarily considered 75 years, but it could be possible that some physicians below this age included in the FNOMCEO list were not at work during the pandemic, as well as some others who were at work even if above 75 years old. It should be considered that many doctors returned to work after retirement to serve the country during the pandemic, and that the usual retirement age for physicians in Italy is currently between 67 and 68 years old, but many of them, also in the non-pandemic era, continue working, perhaps not full time, after formal retirement in case of good health conditions. We also chose 75 years because it was the higher age for which it was clear, at least for some physicians, from the FNOMCEO database that they were at work during the pandemic. When we were not able to retrieve the age of the physicians from the FNOMCEO archive—and this happened in seventeen cases, i.e., 5.8% of the total number of deaths that we analyzed—in case it was not explicitly stated on the webpage that the doctors were no longer on duty, we kept the individual data in the database.

Another important limitation of our study is related to the reporting of the dates of deaths only, for both physicians and the general public. It should be noted that this information is not representative of the dates of infection, especially considering that most of the patients with COVID-19 may have experienced a prolonged treatment process, finally progressing to death after several weeks/months. We tried to partially overcome this limitation by analyzing the death rates based on periods of two months, as reported in Table 2. Furthermore, no information on the specific SARS-CoV-2 variants, associated with different infective characteristics and mortality rates, was available for our analysis. Nevertheless, it should be noted that our results are based on the situation before the spread of the Omicron variant in Italy, which started in November 2021 that substantially changed the pandemic scenario, increasing the numbers of infections, decreasing the effectiveness of the protection of the vaccines against the infection, but also reducing the proportional number of subjects with severe symptoms and deaths compared to the previous variants.

Considering the vaccine dose data, the correlation that we made was with the number of daily doses administered. Unfortunately, no individual data on the types of vaccine administered were available for our analysis, possibly limiting the interpretation of our results. During the first month of the vaccination campaign in Italy, when only the Comirnaty vaccine was approved, and physicians together with other HCW and the most clinically vulnerable individuals in the general public were the target of the vaccination campaign [16], the number of vaccine doses resembled the total number of vaccinated individuals, initially with the first dose, and after 21 days also with the second dose. The longer the time period after the beginning of the vaccination campaign, these data deviate from the number of individual subjects vaccinated, for various reasons: (a) different types of vaccines, with different intervals between the first and second dose; (b) a proportion of subjects who were infected by SARS-CoV-2 and underwent a single vaccine dose; and (c) prolonging the time interval between the first and second vaccine dose up to 42 days after the first few months of the campaign in order to increase the total number of subjects vaccinated with at least one dose [32]. We arbitrarily chose to compare the data on the doses administered with deaths that occurred 30 days after, in order to appreciate both the possible effects of the first dose and of a full vaccination cycle for different types of vaccines, and considering that, in case of infection, the time between symptom onset and death ranges from 2 to 8 weeks [33]. Finally, it should be considered that by comparing the physicians’ deaths with the doses administered in the whole population, we estimated not only the protective effect of the individual vaccination of the doctors but also the protection induced by the increasing number of the general population vaccinated overall, indicating the lower circulation of the virus and/or reduced severity of disease amongst those vaccinated.

## 5. Conclusions

Our analysis of the trends of COVID-19-related mortality amongst Italian doctors during nearly 20 months of the SARS-CoV-2 pandemic has confirmed the large burden in terms of lives lost. In addition, our analysis provides further evidence for previously highlighted risk factors, including the relevant role of age and male sex, and the increased risk observed for general practitioners. On the other hand, our data provide further support for the protective effect of the anti-COVID-19 vaccination campaign: the number of doctors who died in the period March–May 2020, i.e., the beginning of the pandemic in Italy, when the highest rates of COVID-19-related deaths were observed, compared to the same days in March–May 2021, when the majority of the physicians had been vaccinated after the start of the Italian vaccination campaign, was twelve times higher. Moreover, there was a strong inverse correlation between the number of physicians’ deaths and the cumulative number of vaccine doses administered in the Italian population, suggesting both a direct and an indirect protective effect of the vaccines. Finally, the latest available death trends in the period September–October 2021, considering both the physicians and the general public, further confirm the need for a third vaccine dose, currently underway for the majority of the population at risk.

## Figures and Tables

**Table 1:** Physicians’ deaths in Italy from March 2020 to October 2021 according to sex and medical specialty.

		Number of Deaths (%)	Mean Age (±SD)	*p*
**Total**		293 (100)	65.9 (±5.67)	/
Sex	Males	278 (94.9)	66.0 (±5.72)	0.26
	Females	15 (5.1)	64.3 (±4.64)	
Medical specialty	General practitioner	121 (41.3)	66.1 (±4.14)	0.36
	Dentist	22 (7.5)	65.7 (±6.36)	
	Anesthetist/intensivist	16 (5.5)	66.7 (±6.96)	
	Public health doctor	9 (3.1)	62.7 (±4.09)	
	Gynecologist	9 (3.1)	67.8 (±4.23)	
	Cardiologist	8 (2.7)	68.3 (±2.71)	
	Pediatrician	7 (2.4)	68.2 (±3.31)	
	Otolaryngologist	6 (2.0)	65.8 (±8.03)	
	Ophthalmologist	6 (2.0)	64.5 (±4.51)	
	Emergency room doctor	5 (1.7)	58.6 (±12.93)	
	Forensic doctor	5 (1.7)	66.6 (±3.51)	
	Neurologist	5 (1.7)	66.2 (±5.07)	
	Other specialty	57 (19.5)	65.9 (±7.21)	
	Unknown	17 (5.8)	65.9 (±7.33)	

**Table 2:** COVID-19-related deaths amongst Italian physicians and the general population from March 2020 to October 2021.

		Number of Deaths (%)		
		Physicians	General Population	*p*
Total		293 (100)	129,081 (100)	
	Mean age (years)	65.9	80	<0.0001
	Males vs. females (%)	94.9 vs. 5.1	56.5 vs. 43.5	<0.0001
Total number of deaths and vaccination campaign *	Pre-vaccination campaign	239 (81.6%)	98,815 (76.5%)	0.04
	Post-vaccination campaign	54 (18.4%)	30,266 (23.5%)	
Total number of deaths and season **	Warm season	29 (9.9%)	18,478 (14.3%)	0.03
	Cold season	264 (90.1%)	110,603 (85.7%)	
Months	March–April 2020	121 (41.3)	28,908 (22.4)	/
	May–June 2020	11 (3.8)	5287 (4.1)	
	July–August 2020	4 (1.4)	482 (0.4)	
	September–October 2020	10 (3.4)	4433 (3.4)	
	November–December 2020	93 (31.7)	37,474 (29.0)	
	January–February 2021	36 (12.3)	22,231 (17.2)	
	March–April 2021	14 (4.8)	21,990 (17.0)	
	May–June 2021	0 (0.0)	5294 (4.1)	
	July–August 2021	1 (0.3)	1422 (1.1)	
	September–October 2021	3 (1.0)	1560 (1.2)	

* Calculated based on the different starting month of vaccination campaign in Italy for physicians and general public, respectively, in January and March 2021. ** The months from May to October in the years 2020 and 2021 have been considered as the “warm season” in Italy vs. the months from March to April and from November to December in 2020 and the months from January to April in 2021 as the “cold season”.

## Data Availability

The data used for the analysis reported in this paper are publicly available on the official FNOMCEO, ISS and ISTAT websites, as well as in the websites https://ourworldindata.org and https://www.pkegroup.it.
